# Optimizing Photoinitiator Concentration in 3D-Printed Resins for Provisional Dental Restorations

**DOI:** 10.1590/0103-644020256567

**Published:** 2025-11-21

**Authors:** Emanuela Gaviolli, Fabricio Mezzomo Collares, Gabriela de Souza Balbinot, Vicente Castelo Branco Leitune

**Affiliations:** 1 Dental Materials Laboratory, School of Dentistry, Federal University of Rio Grande do Sul, Porto Alegre, RS, Brazil

**Keywords:** Additive Manufacturing, 3D Printing, Stereolithography, Composite Resin, Photoinitiator

## Abstract

The study aimed to evaluate the effect of different TPO photoinitiator concentrations on the mechanical, physicochemical, and biological properties of 3D-printed resins for provisional dental restorations. The resin was formulated with 60 wt.% UDMA, 40 wt.% TEGDMA, 1 wt.% TPO, 0.01 wt.% BHT, and 5 wt.% BaSil. TPO was incorporated at concentrations of 1, 2, and 3 wt.% (TPO1%, TPO2%, and TPO3%, respectively). Specimens were designed using software and printed with an SLA/LCD 3D printer. Post-processing involved ultrasonication in isopropanol followed by UV curing for either 10 or 30 minutes. The resins were evaluated for degree of conversion (DC), flexural strength and modulus, Knoop microhardness, softening in solvent, color stability, and cytotoxicity. No significant difference in DC was observed between TPO1% and TPO3% for both post-polymerization times (p >0.05). TPO3% post-polymerized for 10 minutes exhibited the highest flexural strength and modulus (p <0.05). All groups showed a reduction in hardness after solvent exposure. No statistical difference was found for ΔKHN% (p > 0.05), but post-polymerization time significantly influenced ΔKHN% in the TPO1% group (p < 0.05). Cytotoxicity did not differ significantly among groups (p >0.05). TPO1% post-polymerized for 30 minutes exhibited a color change within clinically acceptable thresholds. The 3D-printed resin with 3% TPO demonstrated significant improvements in mechanical properties, an increased degree of conversion, and no cytotoxic effects. However, it exhibited a significant color change after aging. The 1% TPO group post-cured for 30 minutes showed promising overall results.

## Resumo

O objetivo do estudo foi avaliar o efeito de diferentes concentrações do fotoiniciador TPO nas propriedades mecânicas, físico-químicas e biológicas de resinas impressas em 3D para restaurações dentárias provisórias. A resina foi formulada com 60% em peso de UDMA, 40% em peso de TEGDMA, 1% em peso de TPO, 0,01% em peso de BHT e 5% em peso de BaSil. O TPO foi incorporado em concentrações de 1%, 2% e 3% em peso (TPO1%, TPO2% e TPO3%, respectivamente). Os corpos de prova foram desenhados em um software e impressos com uma impressora 3D SLA/LCD. O pós-processamento envolveu ultrassonicação em isopropanol, seguida de cura UV por 10 ou 30 minutos. As resinas foram avaliadas quanto ao grau de conversão (GC), resistência à flexão e módulo de elasticidade, microdureza Knoop, amolecimento em solvente, estabilidade de cor e citotoxicidade. Não foi observada diferença significativa no GC entre TPO1% e TPO3% para ambos os tempos de pós-polimerização (p >0,05). O grupo TPO3% pós-polimerizado por 10 minutos apresentou a maior resistência à flexão e módulo de elasticidade (p <0,05). Todos os grupos apresentaram redução da dureza após a exposição ao solvente. Não foi encontrada diferença estatística para ΔKHN% (p >0,05), mas o tempo de pós-polimerização influenciou significativamente o ΔKHN% no grupo TPO1% (p <0,05). A citotoxicidade não diferiu significativamente entre os grupos (p >0,05). O grupo com 1% de TPO pós-polimerizado por 30 minutos apresentou alteração de cor dentro dos limites clinicamente aceitáveis. A resina impressa em 3D com 3% de TPO demonstrou melhorias significativas nas propriedades mecânicas, maior grau de conversão e ausência de efeitos citotóxicos. No entanto, apresentou alteração significativa de cor após o envelhecimento. O grupo com 1% de TPO pós-polimerizado por 30 minutos apresentou resultados gerais promissores.

**Figure:**
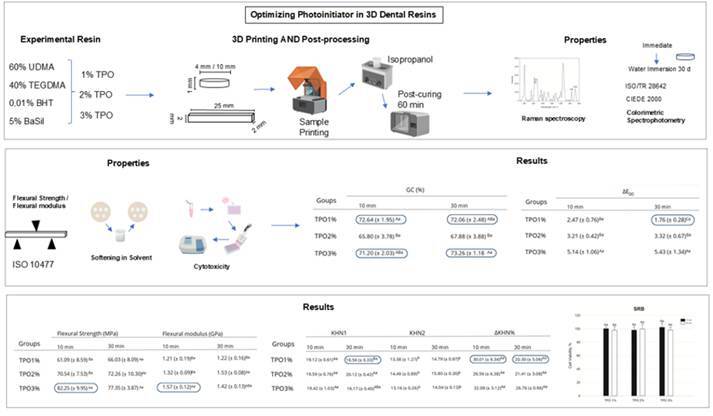

## Introduction

Stereolithography in 3D printing has established itself as the most widely used technique in Dentistry nowadays, standing out for its high precision and resolution ^1^ ^,^ ^20^, as well as the availability of a variety of materials and printers ^3^. Stereolithography technology utilizes photo-polymerizable resins composed of a variety of components, including monomers and oligomers, diluents, inorganic filler particles, and photoinitiators ^4^. These components contribute to enhanced mechanical properties and an increased degree of conversion of the material ^5^. However, to be suitable for printing and intraoral application, these resins must exhibit characteristics such as low viscosity, appropriate refractive index, polymerization capability under ultraviolet or visible light, and biocompatibility ^3^ ^,^ ^4^. For 3D printing of crowns and indirect restorations, materials must achieve a high degree of conversion, excellent mechanical properties to withstand masticatory efforts, favorable aesthetic characteristics, and absence of cytotoxic effects to oral tissues, all determining factors for restoration longevity ^5^.

Another factor contributing to the efficiency of the stereolithography is the variety of light sources used, each with specific wavelength and power characteristics ^6^ ^,^ ^7^. However, the polymerization efficiency of composite resins is strongly influenced by the chemistry of the photoinitiators incorporated in the formulation of these materials ^8^. In this context, the relationship between photoinitiators' absorption spectrum and light emission of the 3D printer is fundamental, as it can directly affect the polymerization process and, consequently, the material’s final performance ^8^. In vat photopolymerization techniques, Stereolithography (SLA) systems employ laser beams with a wavelength of 355 nm. In contrast, Digital Light Processing (DLP) and Liquid Crystal Display (LCD) systems use 405 nm light-emitting diodes (LEDs) ^6^. The use of shorter wavelengths in these printing systems can directly influence the material’s polymerization process and degree of conversion, potentially compromising the material’s properties and the longevity of the restoration ^5^. Thus, there is a need for highly efficient radical polymerization photoinitiators capable of absorbing light within the emission range of these printers ^5^ ^,^ ^6^ ^,^ ^7^ ^,^ ^9^ ^,^ ^10^.

The literature reports a variety of photoinitiators sensitive to ultraviolet and visible light, which are used in photopolymerization systems for 3D printing ^10^. Among the most used photoinitiators in printed resins, diphenyl(2,4,6-trimethylbenzoyl)phosphine oxide (TPO) stands out ^5^ ^,^ ^10^ ^,^ ^11^. This compound, classified as a Norrish Type I Photoinitiator, which does not require interaction with a co-initiator, is recognized for its sensitivity to UV and visible light in the range of 350 to 430 nm when exposed to light ^5^ ^,^ ^9^ ^,^ ^11^. Upon absorbing energy, TPO molecules are elevated to a higher energy level. This is followed by molecular fragmentation, in which one or more chemical bonds are broken within the structure, resulting in the formation of two highly reactive free radicals, R─C^•^(═O) and R─P^•^(═O) ^12^. The free radicals can react with monomers and are responsible for initiating and propagating the formation of the polymer network ^12^. Additionally, TPO's colorless nature facilitates light penetration, enabling deeper polymerization ^5^ ^,^ ^9^, while also reducing the potential for long-term yellowing of the material ^5^. However, it is important to note that at higher concentrations, this Photoinitiator may exhibit cytotoxic effects ^5^.

Different formulations of photopolymerizable resins have been used in 3D printing processes, offering varying chemical and mechanical properties. However, these materials still do not fully meet the desired clinical and performance requirements. Therefore, studies are necessary to test different compositions and concentrations to achieve optimal properties. Considering the importance of the photoinitiation system for the degree of conversion and, consequently, the performance of printed resins, as well as the limitations of 3D printers in terms of light intensity and wavelength, research is being conducted to improve resin formulations and investigate their potential effects on material properties ^5^ ^,^ ^10^ ^,^ ^11^. Thus, this study aimed to evaluate the effect of different TPO photoinitiator concentrations on the mechanical, physicochemical, and biological properties of 3D-printed resins for provisional dental restorations.

## Materials and methods

### Experimental photopolymerizable resin formulation

The experimental photo-polymerizable resin was formulated by mixing 60 wt.% of Diurethane dimethacrylate (UDMA) and 40 wt.% of Triethylene glycol dimethacrylate (TEGDMA). As a polymerization inhibitor, 0.01 wt.% of Hydroxytoluene butylated (BHT) was added. Silanized barium glass at 5 wt.% was added as an inorganic filler. Diphenyl(2,4,6-trimethylbenzoyl)phosphine oxide (TPO) was used as a photoinitiator system at 1, 2, and 3 wt.% to formulate the groups: TPO1%, TPO2%, and TPO3%, and post-polymerization for 10 or 30 minutes, respectively ^13^. All reagents were purchased by Aldrich Chemical, St Louis, MO, USA (Table 1).

**Table 1:** The Components of experimental resin.

Component	Content	Purposes
UDMA	60 wt.%	Organic Matrix
TEGDMA	40 wt.%	Organic Matrix
TPO	1, 2, or 3 wt.%	Photoinitiator
BHT	0.01 wt.%	Inhibitor
BaSil	5 wt.%	Inorganic Filler

The silanization was performed with a 95 wt.% acetone: 5 wt.% 3-(trimethoxysilyl)propyl methacrylate mixture (MPTS). Particles were immersed in the mixture and kept at 37 °C for 24 h for acetone evaporation. The surface of treated particles was chemically characterized by Fourier transform infrared spectroscopy (FTIR - VERTEX 70, Bruker Optics, Ettlingen, Germany) equipped with an attenuated total reflectance (ATR) device (Platinum ATR-QL; Bruker Optics, Ettlingen, Germany). Particles were dispensed over a diamond crystal of ATR. The range of the analysis was 400-3000 cm^−1^ at a 4 cm^−1^ resolution.

To formulate the experimental resin, UDMA and TEGDMA were hand-mixed for 5 min and ultrasonicated for 480 s. TPO and BHT were added and hand-mixed for 5 min and ultrasonicated for 480 s. The BaSil filler particles were added and mixed in a stirrer (SpeedMixer® FlackTek, USA) at a constant speed of 2500 rpm for 20 s.

### 3D printing

For the 3D printing process, an Anycubic Photon Mono 4K printer (ANYCUBIC, China) based on SLA/LCD technology was used, operating at a light wavelength of 405 nm. Different sizes and geometries of specimens were designed using the 3D Builder software version 18.0.1931.0. The generated stereolithography (.stl) files were sliced using Chitubox V.1.9.4 software and transmitted to the 3D printer. The resolution was set at 50 µm thickness for each layer, and an exposure time of 5 seconds was applied to each layer. Upon completion of printing, the specimens were washed with isopropyl alcohol in an ultrasonic tank (L100; Schuster, Santa Maria, RS, Brazil) for 960 s, removing excess uncured resin. Subsequently, post-polymerization processing was carried out using a UV post-curing equipment (Formlabs Inc., Somerville, EUA) with a light wavelength of 405 nm. Two different post-polymerization times were evaluated: 10 or 30 minutes, without any additional heating. Finally, the specimens were finished using grit sandpaper with a sequence of 600 and 1200, respectively.

### Experimental photopolymerizable resin properties - Degree of Conversion

Five disks (n=5) with a diameter of 4 mm and a thickness of 1 mm were 3D printed to determine the degree of conversion (DC). The analysis was performed using Raman spectroscopy (Senterra, Bruker Optics, Ettlingen, Germany). The samples were analyzed using the following parameters: a 785 nm diode laser with 100 mW and a spectral resolution of ~ 3-5 cm^-1^. Accumulation time per spectrum was 10 s with 10 co-additions. One spectrum of unpolymerized resin was obtained using the same parameters. Following the acquisition of the spectra, the peaks corresponding to the bands at 1640 cm^−1^ and 1720 cm^−1^ were analyzed. The degree of conversion was calculated using the following formula:

DC (%) = (1- (R polymerized) / (R unpolymerized)) X 100

Where *R* represents the ratio of peaks between 1640 cm^−1^ and 1720 cm^−1^ of polymerized and unpolymerized resin.

### Experimental photopolymerizable resin properties - Flexural Strength and Flexural Modulus

Flexural strength and flexural modulus were assessed following ISO 10477 ^14^. For each group, five rectangular specimens (n=5) with dimensions of 25 mm x 2 mm x 2 mm were 3D printed. Prior to testing, the specimens were immersed in distilled water at 37 ºC for 24 hours. Flexural strength was determined with a three-point test at a crosshead speed of 1 mm/min in a mechanical testing machine (EZ-SX - Shimadzu, Kyoto, Japan) until the specimens fractured, flexural strength was calculated from the following equation:

$$\sigma \;=\;3Fl\;/\;2{bh}^{2}\;$$

where *F* is the maximum load exerted on the specimen, *l* is the distance (mm) between the supports ± 0.01 mm, *b* is the width (mm) of the specimen immediately before testing, and *h* is the height (mm) of the specimen measured with a digital caliper immediately before testing.

For the flexural modulus (*Ɛ*), the values were obtained by the following equation:

$$Ɛ\;=\;{LS}^{2}3\;/\;4{WH}^{2}3d$$

where *L* is the maximum load, *S* is the span, *W* is the width of the specimen, *H* is the height of the specimen, and *d* is the deflection corresponding to the load *L*.

### Experimental photopolymerizable resin properties - Softening in Solvent

For each experimental group, five printed discs (n=5) measuring 4 mm in diameter and 1 mm in thickness were embedded in acrylic resin and polished (Model 3v, Arotec, Cotia, SP, Brazil) with a felt disc embedded in alumina suspension (Alumina 1.0 µm, Arotec, Cotia, SP, Brazil). The initial Knoop microhardness (KHN1) of the specimens was measured using a digital microhardness tester (HMV 2, Shimadzu, Tokyo, Japan). Five indentations were performed in each specimen with a 50 g load for 15 s. Specimens were then immersed in ethyl alcohol 70% for 2 h, and a new hardness measurement was performed (KHN2). To analyze softening solvent effects, the differences between KHN1 and KHN2 were utilized to calculate ΔKHN%.

### Experimental photopolymerizable resin properties - Colorimetric Spectrophotometry

Color measurements were conducted using a reflectance spectrophotometer (CARY 5000 UV-Vis-NIR; Agilent, Santa Clara, US) equipped with a DRA-1800 integrating sphere. For each experimental group, five 3D-printed disks (n=5) with dimensions of 10 mm in diameter and 1 mm in thickness were prepared. To define the analysis area, a dark mask with an opening of 6 mm in diameter was placed over the surface of each specimen. The color of each specimen was measured and quantified in terms of three coordinate values (L*, a*, and b*), according to the CIELab system (Commission Internationale de l'Eclairage). The analysis was performed at two different stages: immediately after printing and after aging through storage in distilled water. Color difference calculations based on ΔE_00_ (CIEDE 2000) were employed to interpret the degree of perceptibility and acceptability of color changes by ISO/TR 28642 ^15^.

### Experimental photopolymerizable resin properties - Aging by storing in distilled water

Five 3D printed disks (n=5) with dimensions of 10 mm in diameter and 1 mm in thickness were prepared for each experimental group. The specimens were stored in distilled water at 37 ºC for 30 days ^16^ ^,^ ^17^. Following the aging process, the specimens were dried and subjected to color evaluation using colorimetric spectrophotometry.

### Experimental photopolymerizable resin properties - Cytotoxicity

To evaluate the cell viability of gingival fibroblasts on the experimental photopolymerizable resin, the Sulphorhodamine B assay (SRB) was employed. Cells were collected from human gingival tissue after biopsy (approved by the Research Ethics Committee nº 5.969.764). Cells were cultivated in Dulbecco's Modified Eagle Medium (DMEM) and kept at 37 ºC for 24 h. The medium was changed every 2 - 3 days. Before cell culture, five samples (n=5) with a diameter of 4 mm and a thickness of 1 mm were immersed in 1 mL of supplemented DMEM and kept in a 37 ºC incubator for 24 h. For cell treatment, gingival fibroblasts (4 × 10^4^) were seeded in 96-well plates, and after 24 h conditioned media were used to treat cells for 72 h. Cell treatments were performed in triplicate. Cells were fixed and stained with 50 µl SRB 0.4% (Sigma Aldrich, St. Louis, EUA). Cells were suspended in 10% Tris for quantification at 560 nm in a Microplate Spectrophotometer (Multiskan GO, Thermo Fisher Scientific, USA). To normalize the percentage of viable cells, medium without treatment was used as a control.

### Statistical analysis

The normal distribution of the data was assessed using the Shapiro-Wilk test. Statistical analysis for degree of conversion, flexural strength, flexural modulus, KHN1, ΔKHN%, ΔE_00_, and cytotoxicity was performed using Two-way ANOVA and Tukey’s multiple comparisons tests. For comparison between initial and final microhardness (KHN1 and KHN2), a paired Student’s t-test was employed. All statistical tests were performed with a significance level set at 5%.

## Results

Table 2 shows the results of the degree of conversion (DC). TPO1% showed no statistical difference from the TPO3% when evaluated after 10 (p =0.68) or 30 (p =0.77) minutes of post-polymerization. TPO2% showed the lowest conversion degree values at both post-polymerization times. The degree of conversion ranged from 65.80 (±3.78)% to 73.26 (±1.18)%.

**Table 2:** Mean and standard deviation of the degree of conversion (DC), flexural strength, and flexural modulus.

*Groups*	DC (%)		Flexural Strength (MPa)		Flexural Modulus (GPa)	
	10 min	30 min	10 min	30 min	10 min	30 min
TPO1%	72.64 (± 1.95)^Aa^	72.06 (± 2.48)^ABa^	61.09 (± 8.59)^Ba^	66.03 (± 8.09)^Aa^	1.21 (± 0.19)^Ba^	1.22 (± 0.16)^Ba^
TPO2%	65.80 (± 3.78)^Ba^	67.88 (± 3.88)^Ba^	70.54 (± 7.53)^ABa^	75.26 (± 10.30)^Aa^	1.32 (± 0.09)^Ba^	1.53 (± 0.08)^Ab^
TPO3%	71.20 (± 2.03)^Aa^	73.26 (± 1.18)^Aa^	82.25 (± 9.95)^Aa^	77.35 (± 3.87)^Aa^	1.57 (± 0.12)^Aa^	1.42 (± 0.13)^ABa^

Different small letters indicate the statistical difference in the same row between different times in the same group (p < 0.05). Different capital letters indicate the statistical difference in the same column (p < 0.05).

The flexural strength and flexural modulus results are presented in Table 2. The mean values of flexural strength ranged from 61.09 to 82.25 MPa. For the flexural modulus, the mean values ranged from 1.21 to 1.57 GPa. TPO 3% evaluated with 10 minutes of post-polymerization showed the highest mean flexural strength value (82.25 ±9.95) MPa, presenting statistical difference of 1% after 10 minutes of post-curing (p =0.002), but no difference from the TPO2% after 10 minutes of post-curing (p =0.08). No statistically significant difference was observed between TPO concentrations at 30 minutes of post-polymerization (p >0.05). For the flexural modulus, TPO3% post-polymerized for 10 minutes showed a statistical difference with TPO1% and 2%, after 10 minutes of post-curing, presenting the highest mean value (1.57 ±0.12) GPa. TPO2% evaluated with 30 minutes of post-polymerization showed a statistical difference from the TPO1% after 30 minutes of post-curing (p =0.005), but no difference from the TPO3% after 30 minutes of post-curing (p =0.42).

The Knoop microhardness and the percentage of softening in solvent are shown in Table 3. No statistical difference was observed in KHN1 between TPO concentrations when they were evaluated 10 minutes post-polymerization (p >0.05). TPO2% post-polymerized for 30 minutes presented a higher KHN1 value, showing statistical significance from the TPO1% after 30 minutes of post-curing (p <0.05), but showed no statistical difference from the TPO3% after 30 minutes of post-curing (p >0.05). All concentrations of TPO showed a reduction in hardness after immersion in solvent (KHN2) (p <0.05), but without a statistical difference between post-polymerization times. The concentration of Photoinitiator showed no statistically significant influence on the softening in solvent (ΔKHN%) (p >0.05). There was a significant influence of the post-polymerization time on ΔKHN% for TPO1% (p <0.05).

**Table 3:** Softening solvent results. The ΔKHN% was calculated based on the difference between microhardness before and after immersion in the solvent.

*Groups*	*KHN1*		*KHN2*		*ΔKHN%*	
	10 min	30 min	10 min	30 min	10 min	30 min
TPO1%	19.12 (± 0.61)^Aa^	18.56 (± 0.33)^Ba^	13.38 (± 1.27)^b^	14.79 (± 0.97)^b^	30.01 (± 6.34)^Aa^	20.30 (± 5.06)^Ab^
TPO2%	19.59 (± 0.76)^Aa^	20.12 (± 0.42)^Aa^	14.49 (± 0.89)^b^	15.80 (±0.30)^b^	26.56 (± 6.38)^Aa^	21.41 (± 3.08)^Aa^
TPO3%	19.42 (± 1.03)^Aa^	19.17 (± 0.40)^ABa^	13.16 (± 0.26)^b^	14.04 (± 0.13)^b^	32.08 (± 3.12)^Aa^	26.76 (± 0.96)^Aa^

Different small letters indicate the statistical difference in the same row between different times in the same group (p < 0.05). Different capital letters indicate the statistical difference in the same column (p < 0.05).

**Figure 1:**
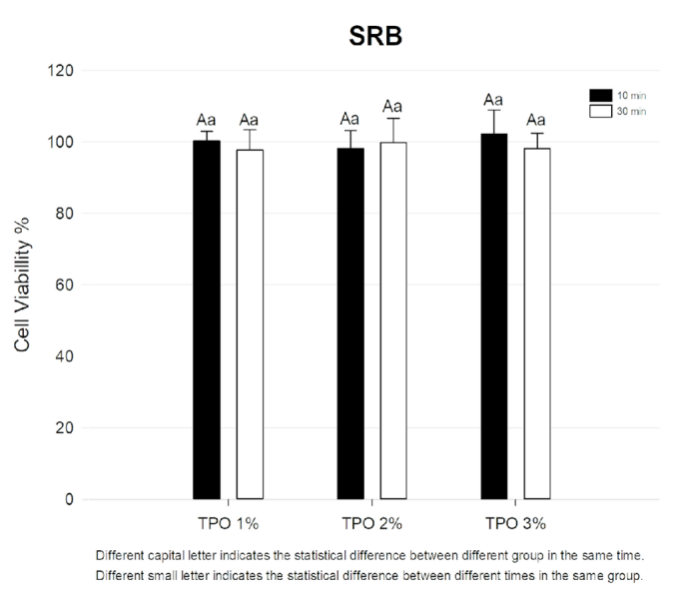
Cell viability of formulated resin. SRB assays were used to assess the % of viable cells after 72 h of the gingival fibroblast cell cultures.

The results of cell viability are described in Figure 1. There was no statistical difference between the groups in the SRB analysis (p >0.05), with the percentage of cell viability ranging from 97.78% to 102.16%.

Table 4 presents the color coordinates' values before and after aging in water, along with the corresponding color results. TPO3% evaluated with 10 minutes of post-polymerization showed a statistical difference from the TPO1% and 2% after 10 minutes of post-curing. All groups showed color change values above the clinical perceptibility threshold (perceptibility threshold = 0.81). TPO1% post-polymerized for 30 minutes showed a color change (1.76) within clinically acceptable thresholds (acceptable thresholds = 1.77).

**Table 4:** Change in color coordinates. Mean and standard deviation of ΔE_00._

	Immediately			Water Immersion			Immediately			Water Immersion			Color Difference ΔE_00_	
*Groups*	10 min			10 in			30 min			30 min			10 min	30 min
	L*	a*	B*	L*	a*	b*	L*	a*	b*	L*	a*	b*	ΔE_00_	
TPO1%	41.0	-0.52	-0.70	46.0	-0.10	-0.19	41.30	-0.53	-1.09	44.74	0.50	-1.88	2.47 (± 0.76)^Ba^	1.76 (± 0.28)^Ca^
TPO2%	36.79	-0.55	1.02	41.89	-0.63	-1.24	36.24	-0.58	0.89	41.49	-0.43	-1.38	3.21 (± 0.42)^Ba^	3.32 (± 0.67)^Ba^
TPO3%	33.38	-0.44	0.67	44.50	-1.07	-0.86	32.66	-0.40	1.43	43.15	-0.60	-1.27	5.14 (± 1.06)^Aa^	5.43 (± 1.34)^Aa^

Different small letters indicate the statistical difference in the same row between different times in the same group (p < 0.05). Different capital letters indicate the statistical difference in the same column (p < 0.05).

## Discussion

Photosensitive initiators perform a fundamental role in the photopolymerization process. The photoinitiators determine the reaction mechanism through the interaction between the light absorption characteristics of the Photoinitiator and the emission spectrum of the light source ^5^. With the advancement of LED-based light source technology for 3D printers, a wide variety of photoinitiators have been developed for compatibility with these sources. The concentration of photoinitiators can affect the polymerization process, thereby influencing the material's properties and long-term stability ^10^. In this study, the Photoinitiator TPO was incorporated into an experimental photo-polymerizable resin for 3D printing at different concentrations to evaluate its influence on the material’s performance. The results of this study showed that using TPO at a concentration of 3% combined with 10 minutes of post-curing significantly improved the mechanical properties of the material, increased the degree of conversion, and did not exhibit cytotoxicity. However, this group showed a significant color change after aging in distilled water. The group containing 1% TPO and post-cured for 30 minutes presented promising overall results.

Considering that 3D printers operate with UV light wavelengths ranging from 355 to 405 nm ^6^ ^,^ ^9^, the initiator must be sensitive to this range. In other words, it should efficiently generate free radicals with lower energy input by absorbing light at shorter wavelengths ^9^. In this context, TPO stands out as a widely employed photoinitiator in 3D printing systems. It is a Norrish type I molecule ^5^ ^,^ ^11^, known for its ability to absorb light within the 350 and 430 nm range. Moreover, it does not require co-initiators, offering notable advantages such as a high polymerization rate and color stability ^18^. The concentration of the Photoinitiator can influence the degree of conversion of the material, and incomplete polymerization may lead to changes in properties over time ^10^. In this study, no significant differences in the degree of conversion were observed between the TPO1% and TPO3% groups. Additionally, post-polymerization time did not significantly affect the degree of conversion. These results suggest a proper correlation between the absorption characteristics of the Photoinitiator at these concentrations and the emission spectrum of the light source used in this study ^5^.

The observed results for the high degree of conversion achieved at TPO concentrations of 1 and 3% can be attributed to the efficiency of TPO initiation, which produces two free radicals per molecule. These free radicals initiate the polymerization process and become covalently integrated into the polymer matrix, demonstrating high reactivity. This characteristic makes TPO particularly effective even at lower concentrations ^11^. Additionally, TPO demonstrates efficient light absorption, with a peak absorption between 380 and 400 nm. Its absorption curve extends into the blue region, aligning well with the wavelengths commonly used by 3D printers ^11^.

In photo-polymerizable resins, the choice of the type and concentration of the Photoinitiator, as well as the resulting degree of conversion, are critical factors that significantly influence the mechanical strength of printed objects. Efficient polymerization is essential, as it reduces the amount of residual free monomers that could otherwise react, thereby minimizing potential cytotoxic effects on tissues and enhancing the mechanical performance of the material ^13^. Appropriate mechanical properties are fundamental in restorative materials, particularly in areas subjected to significant masticatory stress ^5^. In this study, as the concentration of TPO increased, mechanical properties such as flexural strength and flexural modulus improved. The TPO3%, evaluated after 10 minutes of post-polymerization, was more effective than the TPO1% in enhancing the material’s mechanical properties. TPO 2% may have exhibited a lower degree of conversion compared to TPO 1% after 10 minutes of post-polymerization, possibly due to differences in crosslink density and the quality of the polymer network formed. In contrast, the post-polymerization time did not significantly affect the mechanical properties.

The improvement in the material’s mechanical properties with the use of higher photoinitiator concentrations may be attributed to increased monomer crosslinking, as the mechanical properties of a resin depend on the density of crosslinks and the quality of the polymer network formed during polymerization ^19^. In this context, a greater number of photoinitiator molecules can enhance light energy absorption, leading to higher excitation of ions and increased monomer crosslinking ^5^. This process contributes to greater hardness and strength in the resulting polymer.

In this study, TPO concentration did not significantly affect Knoop microhardness (KHN1), except for the TPO1% post-polymerized for 30 minutes, which showed a difference compared to TPO2% after 30 minutes of post-curing, presenting a lower KHN1 value. These findings may be attributed to the interaction between the organic and inorganic matrix ^20^. The presence of organic compounds on the surface of filler particles may reduce reactivity and wetting ability in those areas, thereby influencing particle dispersion and their interaction with the resin matrix ^21^. Although no significant differences were observed among the groups regarding the percentage of degradation (ΔKHN%), all groups exhibited a decrease in Knoop microhardness after immersion in alcohol (KHN2). The literature suggests that solvent exposure promotes plasticization of the polymeric network, weakening the interactions between polymer chains and facilitating the leaching of unreacted monomers. This, in turn, leads to reduced material hardness ^17^. This effect may be related to the quality of the polymer formed, including crosslink density and network porosity ^17^. Light exposure time and intensity can influence the crosslinking density, thereby optimizing the mechanical properties, clinical performance, and biocompatibility of the materials ^16^. However, these factors are directly linked to the type of printer and the material composition. Additionally, solvent-induced degradation may also be influenced by the material's formulation and the interaction between its organic and inorganic components ^17^.

Concerns regarding the cytotoxicity of photopolymerizable resins stem from the potential release of residual monomers and photoinitiators into the oral environment ^5^ ^,^ ^11^. In this study, there was no significant difference among the groups for cytotoxicity, and all groups showed high values of cell viability. These findings may be attributed to the high degree of conversion of the experimental resin, indicating that the polymerization efficiency of TPO is reliable for 3D printing resins. Moreover, TPO becomes chemically integrated into the polymer network and is not completely leached after polymerization. However, previous studies have reported that TPO exhibits concentration-dependent cytotoxicity; therefore, its use at high concentrations is not recommended for clinical applications ^5^. In the present study, up to 3 wt.% of TPO did not increase the cytotoxicity of the composite resin.

Color stability is a relevant factor in material selection and the longevity of dental applications ^5^ ^,^ ^15^. TPO is a photoinitiator with a lighter shade, which may help reduce issues related to color instability in materials ^5^. In this study, color stability was assessed using the CIEDE 2000 metrics ^15^ ^,^ ^22^. Groups with higher photoinitiator concentrations exhibited more yellowish hues initially but became lighter after aging in distilled water. This type of color change can be influenced by the material’s characteristics and the staining capacity of the immersion solutions. Distilled water can cause discoloration depending on the material’s hydrophilicity and the degree of water absorption by the resin matrix ^23^. This change can be observed in Table 4, where the b* values decreased after water immersion, indicating a shift toward negative b* values. This reflects a reduction in yellow intensity, resulting in a lighter appearance post-immersion. Water sorption can affect material translucency and color, often leading to a lightening effect after absorption ^24^. The organic matrix composition influences water uptake; specifically, the high TEGDMA concentration in the experimental resin used here may produce a polymer network more susceptible to hydrosolubilization ^17^. A reduction in yellow chroma toward the blue region of the color spectrum has been reported in other studies involving photoinitiators with light absorption in the UV wavelength range ^5^. In this study, all groups showed color changes, as observed in Table 4. The clinical acceptability threshold for ΔE_00_ is 1.77, while the perceptibility threshold for ΔE_00_ is 0.81 ^15^ ^,^ ^22^. Only TPO1% with 30 minutes of post-polymerization showed a color change within clinically acceptable thresholds.

There are still limitations associated with photopolymerizable resins used in 3D printing, including challenges to wear and impact resistance, color stability, long-term degradation, and dimensional accuracy. These limitations hinder the production of functional components that can maintain durability in the oral environment ^2^ ^,^ ^25^. Therefore, optimizing the photopolymerization process for resins used in 3D printing depends on the appropriate combination of wavelength, light exposure time, and the type of Photoinitiator, critical factors that directly influence the mechanical properties, biocompatibility, and overall performance of the final material.

The limitations of this study are related to the potential sedimentation and agglomeration of the material during printing, which may affect its properties; therefore, further investigations are recommended. Additionally, the color stability of the material may be influenced by the concentration of the organic matrix in the experimental resin. Future studies should explore this aspect, as well as the need for longitudinal studies related to the material’s mechanical properties.

## Conclusion

The 3D printing resin with 3% of TPO demonstrates a significant improvement in the mechanical properties of the material, as well as an increase in the degree of conversion and non-cytotoxicity to cells. However, there was a significant color change after aging. Group with 1% of TPO pos-cured during 30 min presents promising results.
